# Properties of Anion Exchange Membranes with a Focus on Water Electrolysis

**DOI:** 10.3390/membranes12100989

**Published:** 2022-10-12

**Authors:** Hamza Khalid, Malikah Najibah, Hyun S. Park, Chulsung Bae, Dirk Henkensmeier

**Affiliations:** 1 Hydrogen Fuel Cell Research Center, Korea Institute of Science and Technology, Seoul 02792, Korea; 2 Division of Energy and Environment Technology, KIST School, University of Science and Technology, Seoul 02792, Korea; 3 KHU-KIST Department of Converging Science and Technology, Kyung Hee University, Seoul 02447, Korea; 4 Department of Chemistry and Chemical Biology, Rensselaer Polytechnic Institute, Troy, NY 12180, USA; 5 Green School, Korea University, Seoul 02841, Korea

**Keywords:** AEM, mechanical properties, conductivity, alkaline stability, water permeability

## Abstract

Recently, alkaline membrane water electrolysis, in which membranes are in direct contact with water or alkaline solutions, has gained attention. This necessitates new approaches to membrane characterization. We show how the mechanical properties of FAA3, PiperION, Nafion 212 and reinforced FAA3-PK-75 and PiperION PI-15 change when stress–strain curves are measured in temperature-controlled water. Since membranes show dimensional changes when the temperature changes and, therefore, may experience stresses in the application, we investigated seven different membrane types to determine if they follow the expected spring-like behavior or show hysteresis. By using a very simple setup which can be implemented in most laboratories, we measured the “true hydroxide conductivity” of membranes in temperature-controlled water and found that PI-15 and mTPN had higher conductivity at 60 °C than Nafion 212. The same setup was used to monitor the alkaline stability of membranes, and it was found that stability decreased in the order mTPN > PiperION > FAA3. XPS analysis showed that FAA3 was degraded by the attack of hydroxide ions on the benzylic position. Water permeability was analyzed, and mTPN had approximately two times higher permeability than PiperION and 50% higher permeability than FAA3.

## 1. Introduction

Hydrogen produced by water electrolysis with energy from renewable resources is an important component in future energy scenarios. While the energy demand for a few hours (e.g., variation between night and day) could be stored in lithium-ion batteries, storing large amounts of energy from seasonal fluctuations in wind and solar energy needs a new energy storage medium. Among long duration energy storage technologies, the generation of hydrogen using renewable energy and water electrolysis and its storage in the gas grid, tanks and caverns have been suggested as the most attractive option. Furthermore, hydrogen (or in its bound form as liquid organic hydrogen carriers or as ammonia) is one of the most suitable energy carriers to transport renewable energy from countries with excess renewable energy to countries which have limited access to renewable energy.

Water electrolysis traditionally uses alkaline electrolysis systems, which cannot be easily powered by intermittent renewable power sources [1,2]. Proton exchange membrane water electrolysis (PEM WE), which uses a thin, dense membrane (e.g., Nafion) and highly active, corrosion-resistant platinum and iridium catalysts, overcomes some of the shortcomings of alkaline water electrolysis systems [3]. However, for cost and environmental reasons [4,5], perfluorinated Nafion membranes are not the ideal separator. Additionally, the cost and availability of platinum and the even scarcer iridium-based catalysts is seen as a major limitation for broad adoption of PEM WE [6]. An alternative solution could be anion exchange membrane water electrolysis (AEM WE), which uses a thin, dense anion exchange membrane, and potentially earth-abundant non-noble metal catalysts and steel- or nickel-based porous transport layers and flow fields [7]. While the alkaline stability of AEM has been a major hurdle, recent advances in AEM research led to significantly improved membranes. Key strategies are to (1) avoid aromatic ether bonds in the polymer backbone [8], and (2) to tether the quaternary ammonium group with an alkyl spacer, or to use heterocycles with a conformation which prevents Hofmann elimination [9], or imidazolium ions in which the reaction with hydroxide ions is hindered by bulky substituents [10,11]. These strategies resulted in the commercialization of Ionomr’s Aemion membranes, Orion Polymer’s TM1 membranes and Versogen’s PiperION membranes.

For use in AEM water electrolyzers, the membrane should have low gas crossover, high conductivity, high dimensional stability (i.e., low swelling when humidity and temperature increase) and high alkaline stability [12]. Unfortunately, to date, there is no ex situ method which allows analysis of gas crossover under conditions which resemble those of an electrolyzer [13,14]. One reason is that established methods measure the increase in pressure on the low-pressure side of a membrane or the concentration of gas species in a carrier gas on the receiving side of a membrane—in both cases, the membrane is fully in contact with (dry) gases. Another reason is that ions moving through the membrane not only drag water molecules but also gas molecules, so that a significant dependence of the real crossover on the current density is found. Dimensional stability is usually only measured as swelling from dry to wet state. Although this would be important information, it is not usually tested if this swelling is reversible. Therefore, it is not well-known to which extent a decrease in temperature back to room temperature will induce stresses in the membrane. Finally, the mechanical properties of membranes are usually tested only for the dry membranes, and the effect of water and temperature on the mechanical properties is not reported.

In this work, we evaluate several of the above-mentioned properties for some selected membranes. For the first time, the mechanical properties of AEMs were measured by stress–strain tests in the dry state and in water at 30 and 60 °C. The reversibility (i.e., spring-like behavior) of length swelling was analyzed by switching between high temperature and room temperature. Recently, a method to measure the “True hydroxide conductivity” was introduced by Ziv and Dekel [15,16]. The name was coined to stress that the method allows to assess the conductivity of fully hydroxide-exchanged membranes, in contrast to only partially ion-exchanged membranes or membranes which unavoidably became contaminated by CO_2_ during a short time of handling in air. The method was developed for characterizing fuel cell membranes and measurements were conducted in a hydrogen atmosphere. In this work, we measure true hydroxide conductivity of AEM in temperature-controlled water in a modified Ziv–Dekel method [15,17], and the alkaline stability of membranes was measured by monitoring their hydroxide conductivity over the time of 4 weeks. Finally, water vapor permeability was analyzed.

## 2. Materials and Methods

### 2.1. Materials

Fumasep FAA3-50 and FAA-PK-75 (mechanically reinforced) were obtained from Fumatech (Bietigheim-Bissingen, Germany), PiperION 15 (mechanically reinforced) and PiperION 20 (self-supported) were made by Versogen and obtained from Fuel Cell Store (College Station, TX, USA), Nafion 212 was made by Dupont and obtained from FC International (Seoul, Korea). mTPBr, the starting material for Orion Polymer’s TM1, was provided by Prof. Chulsung Bae (RPI, USA) [18]. mTPN and PBI/mTPN were fabricated according to the literature [17]. All the AEMs were received in the bromide form and changed into the chloride form by immersion in 0.5 M NaCl solution for 24 h, followed by immersion in DI water for 24 h to remove any excess salts. Nafion was activated by immersion in 0.5 M HCl.

### 2.2. Mechanical Properties

Mechanical properties were measured on a Cometech QC-508E universal testing machine (Cometech, Taichung City, Taiwan). Each specimen was mounted on grips and pulled to failure at a crosshead speed of 10 mm min^−1^. To obtain the average mechanical properties for every single membrane, the minimum of five successful samples were examined at the same conditions. Dry measurements were performed in ambient atmosphere at RT (23 to 27 °C) between 50 and 70 %rh (rh = relative humidity). The membranes were cut into 4 × 1 cm^2^ strips of rectangular shape, changed into the chloride form as described in 2.1, and were then dried at 60 °C in the vacuum oven for 24 h, before the measurement samples were allowed to equilibrate in lab atmosphere for at least two to three hours. For measurements in wet conditions, a water tank was assembled around the lower, immobile grip. Water was heated externally in a beaker and pumped to the water tank and back with the help of two submersible pumps. Samples were ion-exchanged and washed in DI water, as described above, but not dried. Directly before testing, samples were equilibrated by immersion in water for at least 1 h at the selected temperature. After assembly between the grips, samples were allowed to equilibrate for another 15 min at the selected temperature.

### 2.3. Alkaline Stability

Membranes were cut into 4 × 1 cm^2^ strips and then immersed in 1 M KOH (5.5 wt%) solution using sealed polypropylene vials and stored in an oven at 60 °C. Every 7 days, the solutions were refreshed to ensure that the alkalinity was not reduced by absorption of CO_2_. After 1, 3, 5, 7, 14, 21 and 28 days, solutions were cooled down to room temperature, samples were taken out and washed with DI water for approximately 5 min. Then, in-plane conductivity was measured at room temperature, and the samples were again immersed in KOH solution to continue the stability test. At the end of all tests, the surface chemistry of the samples was examined using X-ray photo-electron spectroscopy (XPS; PHI 5000 VersaProbe; Ulvac-PHI, Chigasaki, Japan) with a monochromatic Al Kα (1486.6 eV) X-ray source.

### 2.4. Swelling Behavior

Membranes were cut into 4 × 1 cm^2^ strips and ion-exchanged as described in Section 2.1. Several samples were dried at 60 °C in the vacuum to obtain the dry length L_dry._ The remaining samples were stored in water, and the temperature of the water was successively changed between a high temperature, then RT, and back to a higher temperature (i.e., 30, RT, 40, RT, 50, RT, 60 °C). At each temperature step, the membrane was allowed to equilibrate for 1 h, and the wet length L_wet_ was measured by transferring samples into a Petri dish containing water of the same temperature and taking a photo of the sample inside the water next to a ruler. For measuring dimensions of transparent membranes, both edges were colored with help of a permanent color marker. Analysis of the photos was conducted with GIMP 2.0 software. Then, the wet thickness T_wet_ was measured with a thickness gauge equipped with disc-shaped sensors (1 cm diameter). The swelling ratio (SR) of the membrane samples was calculated from the difference between the wet and dry dimensions.

(1) $$\mathrm{SR}\;\left(\mathrm{length}\right)=\frac{L_{\mathrm{wet}}-L_{\mathrm{dry}}}{L_{\mathrm{dry}}}\times 100\%\;\mathrm{SR}\;\left(\mathrm{thickness}\right)=\frac{T_{\mathrm{wet}}-T_{\mathrm{dry}}}{T_{\mathrm{dry}}}\times 100\%$$

### 2.5. Conductivity

Wet membrane samples of 4 × 1 cm^2^ size were assembled into a four-electrode in-plane conductivity cell (Bekktech BT-110, Scribner, Southern Pines, NC, USA). The cell was immersed in water, which was constantly de-aerated by bubbling with nitrogen to remove CO_2_. To remove all carbonates from the membranes, a voltage of 2V was applied over the inner electrodes [17]. Every 30 min, electrochemical purging was stopped, and several linear voltage sweeps were conducted over the range of 100 mV to −100 mV with a scan rate of 100 mV/s until the resistance reached a constant value. Conductivity was calculated by the following equation.

$$\mathrm{In-plane}\;\mathrm{conductivity},\;\sigma \;(\mathrm{mS}\;{\mathrm{cm}}^{-1})=\frac{1000d}{R_{\mathrm{mem}}w\cdot t\;}$$

where d represents distance between the voltage-sensing electrodes (cm), w is the width of membrane sample (cm), and t is membrane thickness after immersing in DI water (cm). Set value of d is 0.425 cm, and w was 1 cm.

### 2.6. Water Permeability

Holes of 6 mm diameter were drilled into the screw caps of plastic-capped glass vials. Membrane samples were assembled between the cap and its sealing, so that the hole was covered by the membrane. Then, the vials were filled with water and deposited in an environmental chamber. The assumption made was that the small head space in the vial had a relative humidity of 100%. The outer atmosphere was controlled to 60 °C and a relative humidity of 50%. For 3.5 days, the weight of the vials was measured every 12 h. For each membrane type, 3 samples were tested.

## 3. Results and Discussion

**Figure 1:**
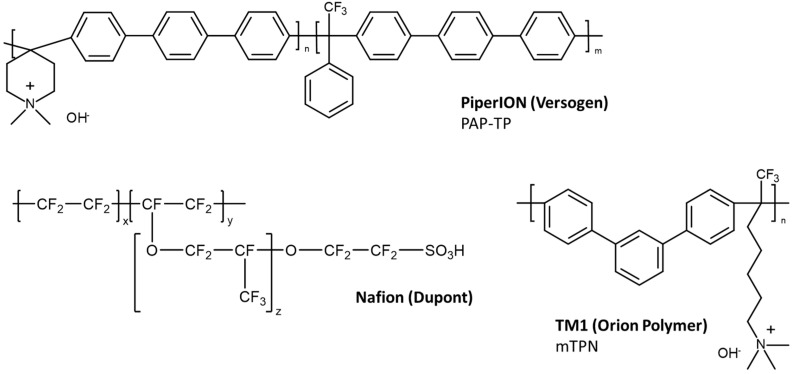
Chemical structures of PiperION (PAP-TP, n = 60–85 [19]), TM1 (mTPN [18]) and Nafion; the structure of Fumatech’s FAA3 is not disclosed in detail, but it is an aromatic ether containing polymer functionalized with quaternary ammonium groups.

Six AEMs were selected for this work: Fumatech’s FAA3-50, Fumatech’s FAA3-PK-75, Versogen’s PI-20 and its reinforced sibling PI-15, self-supporting mTPN (which is the base material of Orion Polymer’s TM1) and PBI/mTPN, in which the ion-conducting mTPN phase is covalently bonded to a PBI nanofiber mat [17]. For comparison, some data were also collected for Nafion 212, which is a cation exchange membrane (CEM). Their specified properties are summarized in Table 1, and the chemical structures, as far as they are disclosed, are shown in Figure 1.

Nafion has the lowest IEC, because the density of perfluorinated materials is much higher than that of hydrocarbon-based polymers. PI-20 has the second highest IEC (2.35 mmol/g), followed by mTPN (2.20 mmol/g) and FAA3 (1.65–2.18 mmol/g). Reinforcement with a porous support reduces the IEC of the membranes. One of the most stable membranes to date seems to be TM1, which is based on mTPN [18], which was found to be the most stable among 50 tested membranes [21].

### 3.1. Mechanical Properties

**Figure 2:**
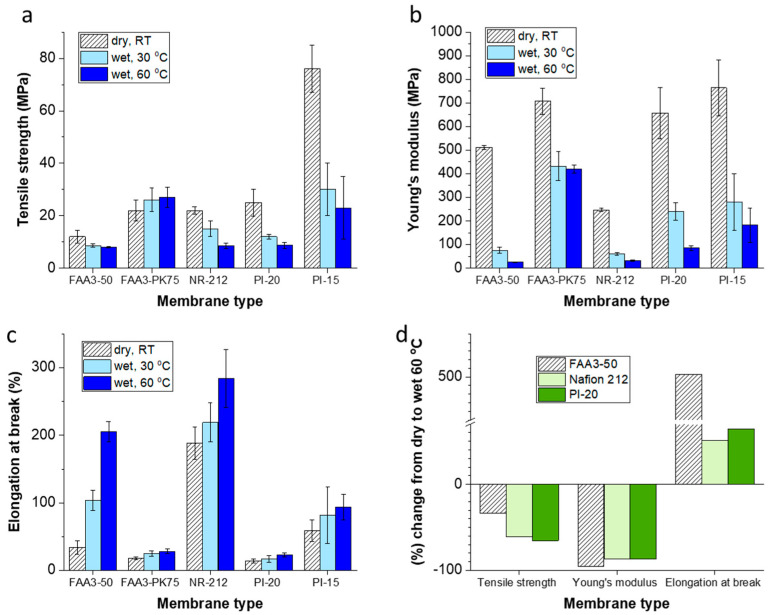
Mechanical properties: (**a**) tensile strength, (**b**) Young’s modulus; (**c**) elongation at break; (**d**) percentage change from dry to wet, 60 °C for non-reinforced membranes.

A modification of the tensile testing machine (see Figure S1) allows to measure stress–strain curves in dry state, in water at 30 °C and in water at 60 °C. As expected, the tensile strength decreases from dry state to wet state, and in the wet state with increasing temperature (Figure 2; exemplary stress–strain curves are shown in Figure S6). The only exception is FAA3-PK-75, which is reinforced by a poly(ether ketone) fiber mesh. In previous work, it was observed for an aged FAA3-PK-75 membrane that it cracked several times before it broke into two separate pieces [17]. Hypothetically, when the FAA3 matrix swells in contact with water, the contact between supporting fibers and matrix intensifies, and prevents stretching or slipping of the fibers. In addition, the elongation at break strongly increases for FAA3-50 with increasing humidity and temperature from <50% in the dry state to >200% at 60 °C in water, so that any local stretching/straightening of the fibers will not lead to cracks. While the elongation at break increased for all tested membranes with temperature and humidity, the Young’s modulus decreased.

Interestingly, PI-20 and Nafion 212 showed similar changes from dry to hot and wet conditions for tensile strength, Young’s modulus and elongation at break, when the data are normalized to 100% in the dry state (Figure 2d). In comparison to these two membranes, FAA3-50 lost less of its original tensile strength, but the elongation at break increased 500%.

Because hydroxide-exchanged membranes absorb CO_2_ from air, the tests were conducted with chloride-exchanged membranes. It is known that hydroxide anions absorb more water molecules into a membrane than chloride anions [20], and the expectation was that hydroxide-exchanged membranes, if tested in inert atmosphere, would absorb more water and, thus, would be more plasticized than chloride-exchanged membranes. However, in previous work, we observed that hydroxide-exchanged membranes showed higher conductivity in DI water than in 0.5 M KOH, because the high ionic strength of the solution leads to an unfavorable change in the osmotic pressure, and, thus, reduced water uptake of the membrane [17]. In 1M KOH, the conductivity again increases, because the efficiency of Donnan exclusion decreases with increasing salt concentration in the solution, and a significant amount of KOH is absorbed, changing the membrane gradually into a kind of diaphragm [22,23]. In the following chapter, it will be shown that hydroxide-exchanged membranes may actually swell less in 1M KOH than chloride-form exchanged membranes in DI water. Therefore, it can be expected that the mechanical properties of chloride-form membranes in DI water will be between those of hydroxide-exchanged membranes in DI water (more plasticized) and 1M KOH (less plasticized).

### 3.2. Swelling Behavior

**Figure 3:**
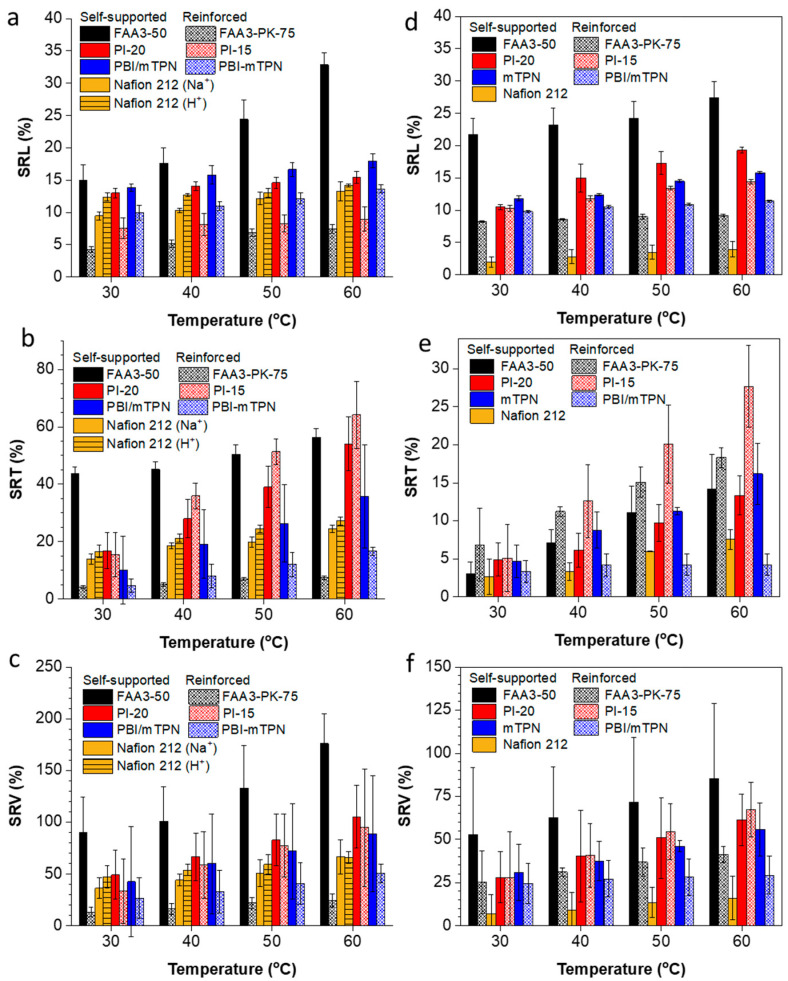
Swelling of membranes in DI water after ion exchange into the chloride form (**a**–**c**) and swelling in 1M KOH after ion exchange into the hydroxide form (**d**–**f**). All samples were punched out from a sheet of the pristine untreated membrane. One set of the samples was dried at 60 °C in the vacuum to measure the dry dimensions, another set of samples was used to measure the wet dimensions consecutively at 30, 40, 50 and 60 °C.

When dry membranes are immersed in water, they swell in length, width and thickness. For melt-extruded membranes, differences between machining and transverse direction are usually observed [24]. For solution-cast membranes, the length and width usually show similar behavior; therefore, only changes in the length and thickness are considered in the following discussion. It can be seen in Figure 3a,d that all reinforced membranes swell less in length than their homogenous analogues. For the thickness direction, the trend is often reversed, because the outer layers are usually not reinforced and, thus, swell freely in thickness, but not in area. As already mentioned and explained in the previous paragraph, the swelling for chloride-exchanged membranes in water could well be higher than that of hydroxide-exchanged membranes in 1M KOH. This effect is clearly seen for the volume swelling (Figure 3c,f).

In fuel cells, membranes experience strong stresses when changes in the humidity of the gas streams reduce or increase the water contents of the membranes. Although membranes swell in all dimensions, the area swelling is considered to be most detrimental for the membrane and leads to wrinkles and cracks at the edge of active and inactive area [25]. At first glance, this problem should be less pronounced in electrolyzers, in which the membranes are always (except for unexpected situations such as failure of a pump) in contact with liquid water. However, the water content also varies with the water temperature, and this raises the concern to which extent membrane dimensions change when the temperature is varied between room temperature and the operating condition of the electrolyzer. Assuming that water absorption does not show hysteresis, Choi and Datta developed a theoretical model to explain the swelling behavior of Nafion which includes a spring constant to account for the forces which counter the water absorption [26]. On the other hand, the strong elongation of real springs leads to irreversible changes. For ion-conducting membranes, this could result in lower dimensional changes from hot to cold than expected when just investigating the change from room temperature to the operating temperature (e.g., 60 °C). Another question is the time scale at which the “spring” goes back to its initial dimension.

**Figure 4:**
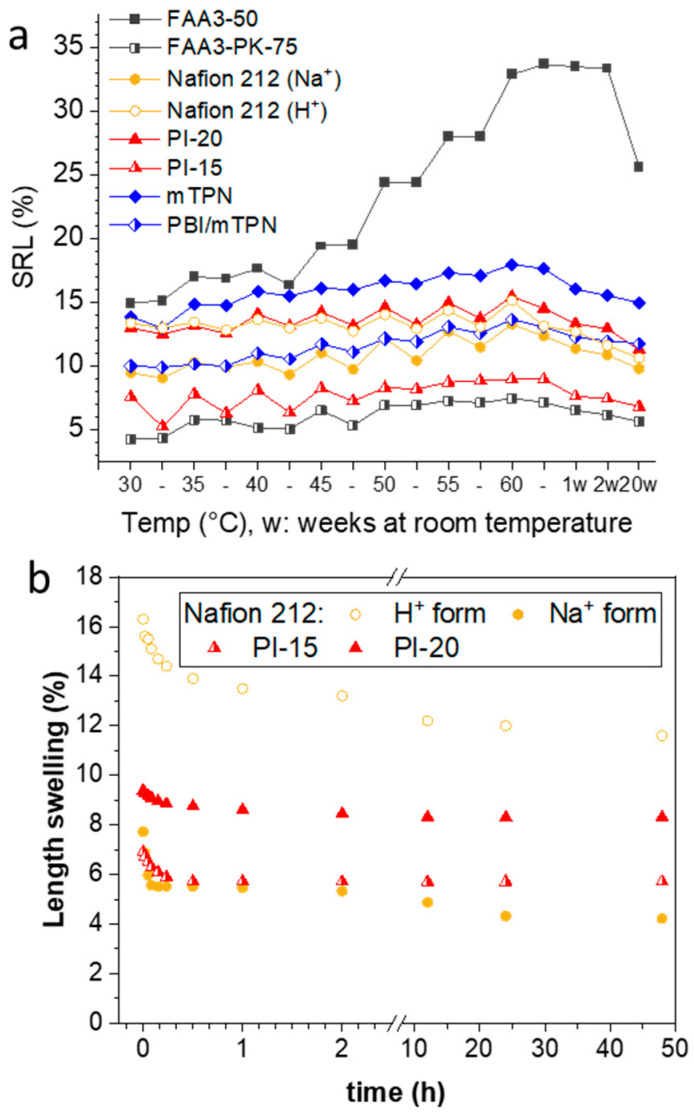
Length swelling in water for chloride-exchanged membranes and its dependence on the water temperature (**a**); the temperature was stepwise increased and decreased again to room temperature (“-“), each step lasted one hour; at the end of the test, the samples were kept at room temperature for 20 weeks (“w”). For FAA3, PI and Nafion (Na^+^) membranes, data were measured in two series (series 1: 30, 40, 50, 60 °C; series 2: 35, 45, 55 °C). (**b**) Shows the length change for membranes which have been stored for 3 h at 60 °C and then are put into water which has been equilibrated at room temperature.

Figure 4a shows the swelling ratio in length for various membranes when the temperature is alternatingly changed from a higher temperature back to room temperature and again to a higher temperature, each time allowing for an equilibration time of 1 h. To some extent, all membranes showed a spring-like behavior at some point; especially, Nafion 212 (Na^+^) and PI-20 showed a spring-like behavior over the whole tested temperature range. The reinforced membranes PI-15 and FAA3-PK-75 only showed a spring-like behavior up to approximately 50 °C, after which the length remained nearly constant. This is because reinforced membranes usually have a layered structure, in which the center layer contains the embedded porous support; the porous support can only be expanded to a certain limit, and after that, further swelling is restricted to the thickness direction (see Figure 3b). FAA3-50 showed strong swelling when the temperature increased, but only a negligible shrink when the temperature decreased again.

After the spring test, the samples were kept in water for 20 weeks. All membranes continuously shrank in length over that time, but still, FAA3-50, FAA3-PK-75, Nafion 212 (Na^+^), PBI/mTPN and mTPN had 1.71, 1.33, 1.03, 1.18 and 1.08 times larger dimensions than those of the initial ones at 30 °C at the beginning of the test (see SRL% of 30 °C vs. 20w in Figure 4a). In contrast to this swelling behavior, PI-15 and PI-20 dimensions became somewhat shorter. However, in the case of PI-15, the length was still longer than the length value at room temperature at the beginning of the test. For PI-20, it should be noted that the thickness swelling at 60 °C was severely pronounced (Figure 3b), and the swelling was highly anisotropic; because oriented morphologies of ion-conducting membranes are usually lost when membranes are highly swollen, shrinking can be expected to occur isotropically, and, thus, the final length could be shorter than the initial one [27,28,29].

A similar “spring” experiment was also conducted with some exemplary membranes in 1M KOH solution (Figure S4). It was found that FAA3-PK-75 and PBI/mTPN show a spring behavior, while the non-reinforced membranes FAA3-50 and mTPN and Nafion 212 did not show a pronounced spring behavior. It appears that the porous support material acts as the main force of the spring behavior, and that KOH plasticizes the ionomers so strongly that it has a weak resilience. As hypothesized at the end of paragraph 3.1, all membranes in Figure S4 increased in length when they were changed from 1M KOH into CO_2_-free water, because of the increased osmotic pressure. Qualitatively, hydroxide-exchanged FAA3-50 and mTPN became very soft and jelly-like in water. It appears necessary that these membranes are reinforced for electrolysis applications.

In another experiment, Nafion 212 (Na^+^), Nafion 212 (H^+^) and PI-15 and PI-20 (both in the chloride form), were immersed in water and heated to 60 °C for 3 h, and then directly transferred to water equilibrated at room temperature (Figure 4b). It can be seen that most length changes are finished within 30 min. The starting values for PI-20 are lower in Figure 4b than in Figure 4a. This could be due to batch-to-batch variations of the membrane, as also discussed later in part 3.4.

### 3.3. Hydroxide Conductivity

In order to measure the hydroxide conductivity of membranes, contact with CO_2_ needs to be excluded. True hydroxide conductivity without any influence of (bi)carbonates can be measured using the Ziv–Dekel method [15]. In this method, AEMs are assembled into a four-point electrode in-plane cell, which is then immersed in a humidity- and temperature-controlled hydrogen gas stream. By applying a current or voltage, water electrolysis at the electrodes leads to formation of hydroxide ions, which then move through the membrane and electrochemically purge out the (bi)carbonates, which then are transported away by the hydrogen gas stream. Due to system limitations and condensation issues, the gas stream is often not humidified to 100 %rh, but to more easily controllable 90 or 95 %rh. For testing the true hydroxide conductivity of AEM in temperature-controlled water, we modified the method [17]. The conductivity cell is immersed in a water-filled beaker, and (bi)carbonates are purged from the membrane by applying a voltage. Bubbling nitrogen through the water transports the released CO_2_ away.

**Figure 5:**
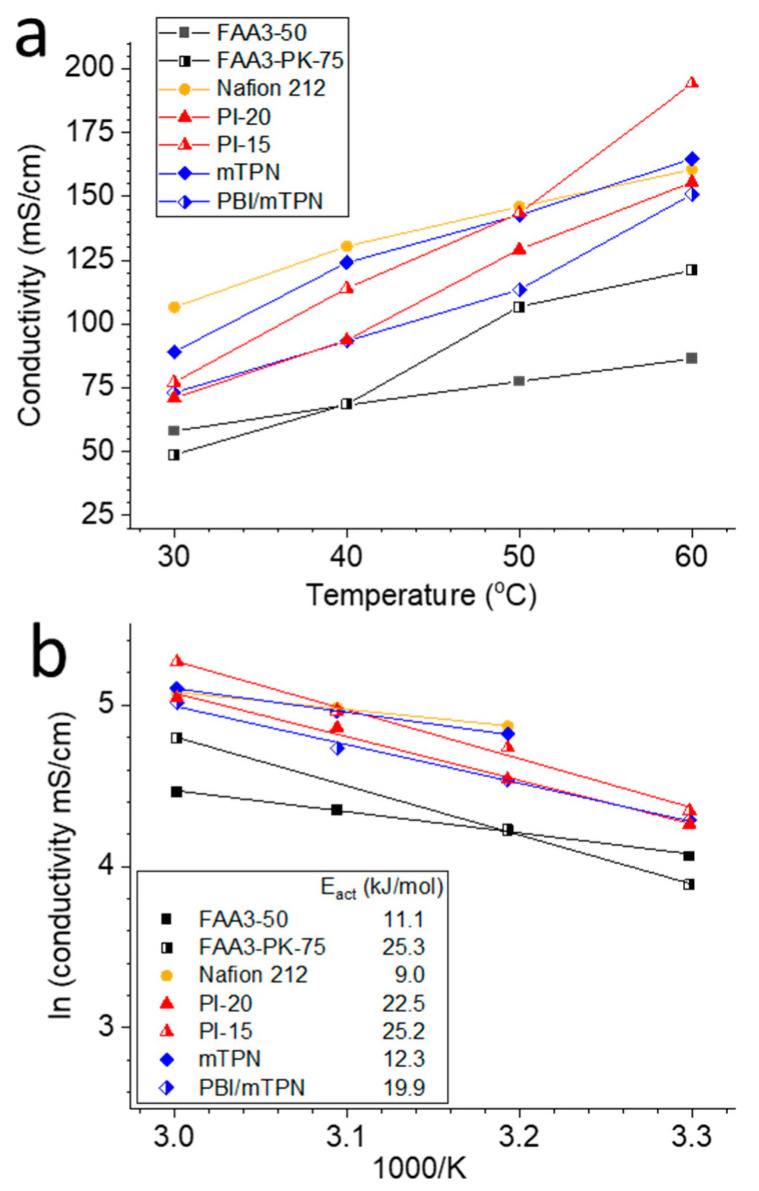
(**a**) Hydroxide conductivity (Nafion: proton conductivity) for different membranes measured at 30, 40, 50 and 60 °C; (**b**) Arrhenius plot; 3 data points which obviously did not match into the linear fit were removed before fitting, and all R^2^ > 0.99.

At 30 °C, Nafion 212 showed the highest conductivity, because the limiting molar conductivity of protons is 1.8 times higher than that of hydroxide ions [30] and Nafion has a very pronounced phase separation (Figure 5a) [31,32]. At 60 °C, which is a relevant temperature for water electrolysis, the conductivity of PI-15 and mTPN was higher than that of Nafion, because the conductivity increase of Nafion is less dependent on temperature than that of the AEMs. This behavior is related to the activation energy of the ionic conduction of H^+^ vs. OH^−^, which can be read out from the Arrhenius plot (Figure 5b). Nafion has the lowest activation energy of 9 kJ mol^−1^, which agrees well with literature data (e.g., 9.5 kJ mol^−1^ in [33]). Interestingly, the activation energies of mTPN (12.3 kJ mol^−1^) and FAA3-50 (11.1 kJ mol^−1^) were similar to that of Nafion. PiperION membranes (PI-20 and PI-15) showed much larger activation energies (23–25 kJ mol^−1^). This seems to be related to the phase separation, which is strongest for Nafion (fluorinated, hydrophobic backbone; ionic groups located at the end of side chains), and water uptake, which is highest for FAA3-50 (see swelling values in Figure 3). In the case of PiperION membranes, the quaternary ammonium groups are located on the rigid backbone of the polymer, which reduces the dissimilarity between the backbone and the ionic moieties, and results in a more amorphous microstructure. Indeed, PiperION was described as a completely amorphous material by Luo et al. [20]. In contrast, the quaternary ammonium group in mTPN is tethered via a C6-alkyl chain to the backbone (Figure 1), and FAA3 has a much less rigid structure due to the ether groups in the backbone. Enhanced phase-separated morphology and presence of bulk-like water will favor conduction via the Grotthus mechanism, which has a lower activation energy than conduction via vehicle mechanism and, thus, will show lower activation energies [34]. As a general trend, the activation energies of the reinforced membranes (i.e., FAA3-PK-75, PI-15, PBI/mTPN) are higher, most probably because the support material limits swelling and, thus, water uptake. The largest difference is seen for FAA3 membranes, which showed the largest difference in swelling and water uptake between self-supporting and reinforced membranes. The very high swelling of FAA3 membranes seen in Figure 3c (nearly 180% volume swelling) could also be the reason why conductivity at 60 °C is higher for the reinforced FAA3-PK-75 membrane. Even though conductivity in general increases with the water uptake due to the better connectivity of the hydrophilic domains and the preference of Grotthus conduction in bulk-like water, an excessive increase in water uptake also reduces the concentration of mobile ions in the hydrophilic phase and, thus, can counteract the conductivity [35].

A large error source is the length and thickness measurement, which has a large influence on the calculated conductivity. In the Ziv–Dekel method, the thickness of wet (bi)carbonate membranes is measured only once at room temperature [15]. However, the real membrane dimensions during the measurement are unknown and will vary with temperature, humidity and ratio of (bi)carbonate and hydroxide. For example, it was reported that FAA3 absorbs approximately 12–13 water molecules per ion in the bicarbonate form, but 18 water molecules per ion in the hydroxide form [20]. Another issue is related to the Schroeder’s paradox; Luo et al. showed that FAA3 has approximately 18 water molecules per hydroxide ion in liquid water, but just 6 when in contact with fully humidified gas streams [21]. For the data shown in Figure 5, the final length of the membranes was measured directly after disassembling the cell. Therefore, the expected trend is that data in Figure 5 is based on lower resistances and larger dimensions than data measured in the original Ziv–Dekel method. For example, FAA3-50 had a conductivity of 68.3 mS cm^−1^ at 40 °C in water, but 10 mS cm^−1^ at 40 °C and 90 %rh was reported for a slightly thinner FAA3-30 membrane in humidified atmosphere [36]. PI-20, which seems to be PAP-TP 80 based on the nominal IEC, was reported to have a conductivity of ca. 125 mS cm^−1^ [19] but had a conductivity of 156 mS cm^−1^ in this work. Presumably, the dimensions used for calculations in that work were based on room temperature values, and, thus, underestimated the real dimensions. Furthermore, it could be possible that ion-exchange reaction to the hydroxide form was not 100% complete in Wang et al.’s work [19], whereas electrochemical purging ensures active removal of other anions. On a similar note: electrochemical purging is not limited to the removal of carbonate anions; it was shown that potassium ions can also be purged from a PEM, resulting in the fully proton-exchanged PEM. Thus, other anions could potentially also be removed if the applied voltage is sufficient [37].

The here reported data are in-plane conductivity, but the performance of actual electrolysis cells will be affected by the through-plane conductivity. While these are often quite similar, they may differ if membranes show a highly anisotropic behavior. On the other hand, the high swelling in hot electrolytes is expected to minimize anisotropy. Furthermore, in-plane conductivity measurements rely less on membrane electrode interfacial resistances than through-plane measurements, for which stacks of 1, 2, 3 membranes need to be measured. Plotting the resistances against the membrane (stack) thickness results in a linear curve; the y-axis intercept displays the cell and interfacial resistances, while the slope can be used to calculate the membrane conductivity [13]. Therefore, in-plane conductivity is usually preferred over through-plane measurements.

### 3.4. Alkaline Stability

**Figure 6:**
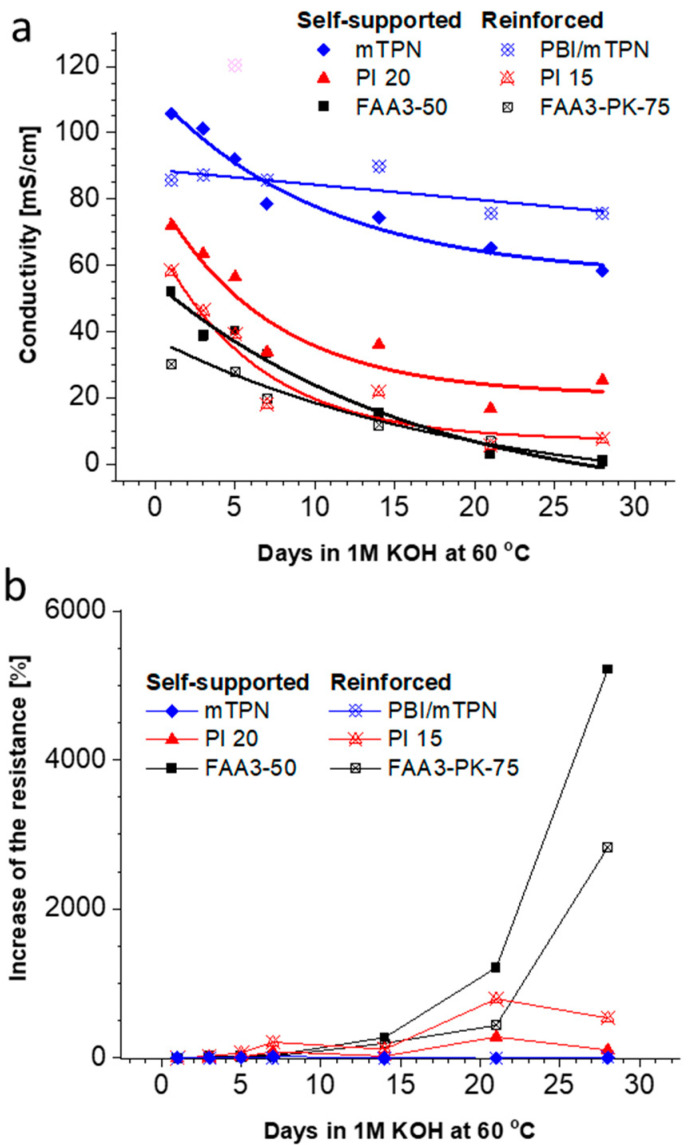
Alkaline stability in 1 M KOH solution at 60 °C; (**a**) changes of the conductivity, lines are exponential fits to guide the eye (the pink data point was excluded from the fitting), (**b**) change of the membrane resistance. The main error source is the exact mechanical thickness measurement of the soft swollen membranes.

For many years, alkaline stability and low conductivity have been the major challenge for AEM development [38,39]. These hurdles have been lowered in recent years by developing membranes which are significantly more conductive and withstand long times in alkaline solutions. Recently, NREL tested over 50 membranes for their alkaline stability by immersing samples for 1000 h in 1M KOH at 80 °C and comparing the chloride-form conductivity before and after the test [21]. Here, we tested the decrease in the true hydroxide conductivity (Ziv–Dekel method, modified to measure in water) by immersing samples at 60 °C in 1M KOH for a certain time, rinsing the membrane in water, testing the conductivity and continuing the test until a total test time of 4 weeks was reached (Figure 6).

The highest initial conductivity was found for mTPN (Orion TM1), followed by PiperION membranes and FAA3 membranes. As expected, the reinforced membranes showed lower conductivities than the parent membranes, because the support materials are not conductive. For all membranes, the degradation data were fitted with an exponential function (Origin). The result confirms the finding of Meek et al. [21], that mTPN (Orion TM1) is one of the most stable materials. Reinforcement with an electrospun PBI nanofiber mat (PBI/mTPN) reduced the degradation further, so that the curve showed a flat, nearly linear trend in the range of −0.02 mS cm^−1^/h. Please note that R^2^ is very low for the linear trend, and that it is possible that the real degradation rate is even lower.

Another representation of the same data is the increase in the resistance (Figure 6b). At the end of the test, the resistance increased 2827% for FAA3-PK-75, 535% for PI-15, 105% for PI-20, 9% for mTPN and just 0.1% for PBI/mTPN.

**Figure 7:**
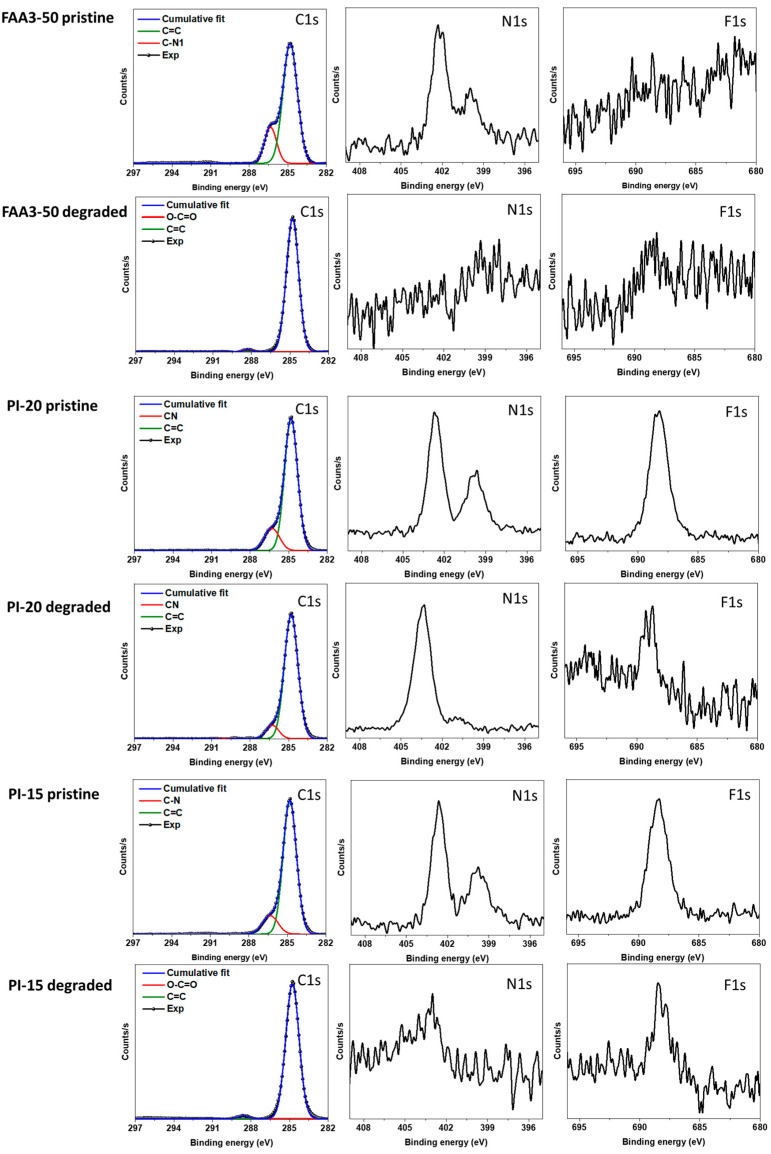
XPS analysis of membranes before and after the stability test shown in Figure 6. Note: samples for pristine PiperION membranes and degraded PiperION membranes were cut from different membrane batches, and it could be that the “pristine” batch was not fully methylated.

The membranes which showed the highest increase in resistance during the alkaline stability test were further analyzed by XPS. The analysis showed that FAA3-50 lost all nitrogen (Figure 7), which nicely explains the very steep increase in resistance. While the exact structure of FAA3-50 is not publicly disclosed, it is known that FAA3 is slightly crosslinked, has an aromatic backbone based on polyphenyleneoxide (PPO) and quaternary ammonium groups linked to the polymer backbone by a benzylic methylene group [40]. Interestingly, the CN-signal around 286.5 eV also vanished during alkaline degradation. This reveals that the degradation of the quaternary ammonium groups occurred by reaction of hydroxide ions with the benzylic position, and not by reaction with the methyl groups (Figure S5).

PiperION PI-20 was much more stable than FAA3-50 in the alkaline stability test, and as expected, XPS analysis still showed large nitrogen signals after the degradation test and CN signals at 286.5 eV. An unexpected observation was that both pristine PI-15 and pristine PI-20 had an additional N1s signal at around 400 eV, which was previously not observed by other researchers [41]. One hypothesis was that the signal could be related to the casting solvent, e.g., acetamide has a signal around 399.6 eV. However, this should be accompanied by a C1s signal around 288.4 eV for the carbonyl group, which was not found [42]. Chen et al. reported that tertiary *N*-methylpiperidinium groups appear at 400 eV [43]. In this case, however, one would expect to find the signal also in the degraded sample. A very simple explanation is that we see batch-to-batch variations, because the commercially obtained membranes had dimensions of 10 × 10 cm^2^, and different membranes were used for the XPS analysis of the pristine membrane and the one tested in the alkaline stability test (see also the different swelling in Figure 4a,b). However, perhaps this result also shows the limitation of the XPS method for membrane analysis: only the surface of samples is analyzed, typically within a depth of 10 nm. Therefore, inconclusive results could be obtained if only the surface of a sample is degraded, or if the composition of the surface can change by re-orientation of the polymer chains. For example, it is known that Nafion energetically favors a hydrophobic surface under dry conditions, and that the polymer chains in the surface layer rearrange in contact with liquid water to form a hydrophilic surface [44,45].

Finally, all nitrogen groups and CN signals vanished for the degraded reinforced PI-15, and a small new signal around 289 eV appeared. This could be one of the above-mentioned surface effects or indicate that the ion conductive material was lost from the surface, so that the reinforcement fibers were analyzed. SEM analysis showed that the membrane morphology still was intact, but SEM EDS indicated that the nitrogen content was very low (Figure S3). One possibility is that the membrane actually survived the alkaline stability test well but then irreversibly lost nitrogen groups after the test, when the membrane was stored in air atmosphere in a dry glass vial; such conditions reduce the number of water molecules around the hydroxide ions, and, thus, promote alkaline degradation. Because all membranes were handled in the same way, this could be an indication that PiperION membranes are nearly as stable as mTPN membranes under humidified conditions, but less stable under dry conditions. This was not further investigated because dry conditions are less relevant for water electrolysis.

### 3.5. Water Permeability

AEM water electrolyzers can be operated in two different ways. One is to feed water to the anode and the cathode. Alternatively, they can be operated with a dry cathode. In the second case, the water consumed in the cathode reaction needs to be replenished by permeation through the membrane from the anode to the cathode side [46]. As a potential disadvantage, the cathode reaction may be limited by water diffusion from anode to cathode, and heat produced in the cathode may increase the temperature. The expected advantages include (1) cost savings of hydrogen production because the cathode product will not be a foam but a gas; thus, the step of separating gas and liquid phase can be omitted; (2) the lower water contents in the produced hydrogen gas could reduce the cost for drying; (3) it reduces the energy losses for pumping.

For efficient “dry cathode” operation, membranes should have a high water permeation, to allow for rapid transport of water from anode to cathode. The diffusion-driven water permeation can be easily measured ex situ by filling a vial with water, sealing it with a membrane, and putting it into a climate chamber to set the humidity on the outer side of the membrane. The humidity on the inner side of the membrane is approximately 100%, if the water level is close to the membrane, and water loss through the membrane, the flux, is measured gravimetrically [47,48].

**Figure 8:**
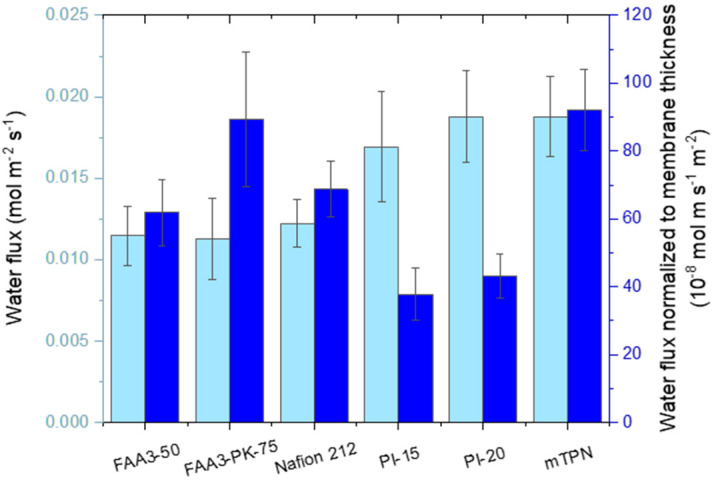
Water flux through chloride-exchanged membranes (Nafion: protonated form) at 60 °C and a relative humidity gradient of 100%/50%.

As shown in Figure 8, Nafion 212 showed a permeability of 68.8 10^−8^ mol m s^−1^ m^−2^, which is in the same range as the value reported by Adachi et al. for Nafion 211 at 70 °C, 100 %rh/50 %rh: 66.2 10^−8^ mol m s^−1^ m^−2^ [49]. PI-20 and mTPN showed the highest fluxes, around 0.0188 mol m^−2^ s^−1^. In terms of material properties, FAA3-PK-75 and mTPN showed the highest permeability, 89.4 and 92.1 10^−8^ mol m s^−1^ m^−2^. The high permeability for FAA3-PK-75 is surprising, because it consists mainly of the same material as the homogenous FAA3-50 membrane, and similar values for the two reinforced membranes were expected. A SEM analysis of reinforced FAA3-PK-75 membranes (Figure S2) revealed that the poly(ether ketone) fibers of the reinforcing fabric are not fully embedded in the center of the membrane and sometimes reach the surface of the membrane, and that voids can form around the fibers. Therefore, the high permeability seems to be the result of water transport along the fibers of the reinforcing fabric. Potentially, these pathways would also lead to increased hydrogen transport over the membrane. However, since the higher thickness of FAA3-PK-75 results in a similar water flux as for FAA3-50, it can be assumed that hydrogen crossover would be similar for this set of two FAA3 membranes.

## 4. Conclusions

Measuring stress–strain curves under dry conditions and in water at 30 or 60 °C showed a similar trend between most membranes, tensile strength and Young’s modulus decreased with increasing humidity and temperature, and elongation at break increased. A large effect was found for FAA3-50, which showed remarkable plasticization at 60 °C, while Nafion showed the least dependence of elongation at break under dry condition vs. at 60 °C in water.

As expected, the length of membranes responded to changes in the water temperature. However, hysteresis was observed. Especially, reinforced membranes tended to keep the swollen dimensions. On the other hand, after heating to 60 °C and cooling down to room temperature again, length shrinking continued for all membranes over the following 20 weeks. This behavior indicates that rapid changes in the temperature will not lead to large immediate stresses for membranes assembled into electrolyzers, but that a slow build-up of stresses could be seen when an electrolyzer is switched off for a longer time.

As a general trend, chloride-exchanged membranes showed a higher volume swelling than hydroxide-exchanged membranes in 1M KOH (because the high ionic concentration in the solution removes water from the membrane), but less than hydroxide-exchanged membranes in water (which absorb water from the solution because of the high ion concentration in the membrane). Therefore, the concentration of the electrolyzer feed will have a large influence on the membrane dimensions and its degree of plasticization.

Mechanical tests and tests for the reversibility of swelling have been conducted for chloride-ion-exchanged membranes only, and not for the more relevant hydroxide form, because of the difficulty to handle membranes in CO_2_ free atmosphere. This could be an item for future work, to get closer to the real conditions in an electrolyzer.

True hydroxide conductivity was measured in a simple set-up, by immersing a standard in-plane conductivity cell in water, removing carbonates from the membrane by the application of a voltage and simultaneously bubbling nitrogen through the water to remove the dissolved CO_2_. The method can be easily implemented in most laboratories. Because membrane dimensions were measured at the end of the test (i.e., when fully swollen at 60 °C and fully exchanged into hydroxide form), and not before the test in the dry state or carbonate-exchanged form, the values tend to be a bit higher than reported by others. It is noteworthy that PI-15 and mTPN showed even higher conductivities than Nafion 212 at 60 °C.

The true hydroxide conductivity method was also applied to monitor the alkaline stability of six different AEMs over a period of 4 weeks in 1M KOH at 60 °C. While the resistance of FAA3 increased several thousand percent, the resistance of PiperION membranes only increased marginally. The most stable membranes were those based on mTPN. XPS analysis revealed that FAA3 degraded by losing all quaternary ammonium groups. Regarding the mechanism, nucleophilic substitution preferentially occurred at the benzylic position, not at the methyl groups.

## Figures and Tables

**Table 1:** Tested membranes and their specifications.

	Dupont Nafion 212	PiperION PI-15	PiperION PI-20	Orion Polymer TM1 (mTPN)	PBI/mTPN	Fumatech FAA3-50	Fumatech FAA3-PK-75
**Type**	CEM	AEM, reinforced with ePTFE	AEM	AEM	AEM, reinforced with a PBI nanofiber mat	AEM	AEM, reinforced with polyetherketone
**Nominal thickness [µm]**	50	15	20	Variable; made by casting from mTPBr [18]	Variable; made according to [17]	50	75
**IEC [mmol/g]**	0.92	na	2.35 ^a^	2.20 ^b^	1.62 ^c^ [17]	1.65–2.18 ^c^	1.23–1.44 ^c^

na: not available; ^a^ presumably hydroxide form [20]; ^b^ OH form, based on chemical structure (Figure 1); ^c^ OH form.

## Data Availability

Data is contained within the article or Supplementary Material.

## Supplementary Materials

The following supporting information can be downloaded at: https://www.mdpi.com/article/10.3390/membranes12100989/s1: Figure S1: Experimental setup for measuring tensile strength in water at controlled temperature with a Universal Testing Machine (UTM). Figure S2: SEM images of FAA3-PK-75. Figure S3: SEM images of PI-15. Figure S4: (a) Length swelling for membranes immersed in 1M KOH solution at different temperatures. (b) Length change for hydroxide exchanged membranes which have been stored for 3 h at 60 °C in 1M KOH and then are put into water which has been equilibrated at room temperature. Figure S5: Degradation pathway of the quaternary ammonium groups in FAA3 membranes. Figure S6: Exemplary stress-strain curves at dry, wet 30 °C and wet 60 °C conditions.
