# Supplementary material for: Properties of Anion Exchange Membranes with a Focus on Water Electrolysis
Source: Membranes (Basel). 2022 Oct 12;12(10):989. doi: 10.3390/membranes12100989 (PMC9609780; doi:10.3390/membranes12100989)
Supplement: Supplementary file 1 [file membranes-12-00989-s001.zip › membranes-1950798-supplementary.pdf]

Supporting Information

Properties of anion exchange membranes with a focus on water electrolysis

Hamza Khalid,[a,b] Malikah Najibah,[a,b] Hyun S. Park,[a,b,c] Chulsung Bae,[d] Dirk Henkensmeier[a,b,e*]

[a] Hydrogen · Fuel Cell Research Center, Korea Institute of Science and Technology, Seongbukgu, Seoul 02792, Republic of Korea

[b] Division of Energy & Environment Technology, KIST School, University of Science and Technology, Seongbukgu, Seoul 02792, Republic of Korea

[c] KHU-KIST Department of Converging Science and Technology, Kyung Hee University, Seoul 02447, Republic of Korea

[d] Department of Chemistry and Chemical Biology, Rensselaer Polytechnic Institute, Troy, NY, 12180, United States

[e] Green School, Korea University, Seoul 02841, Republic of Korea

*henkensmeier@kist.re.kr

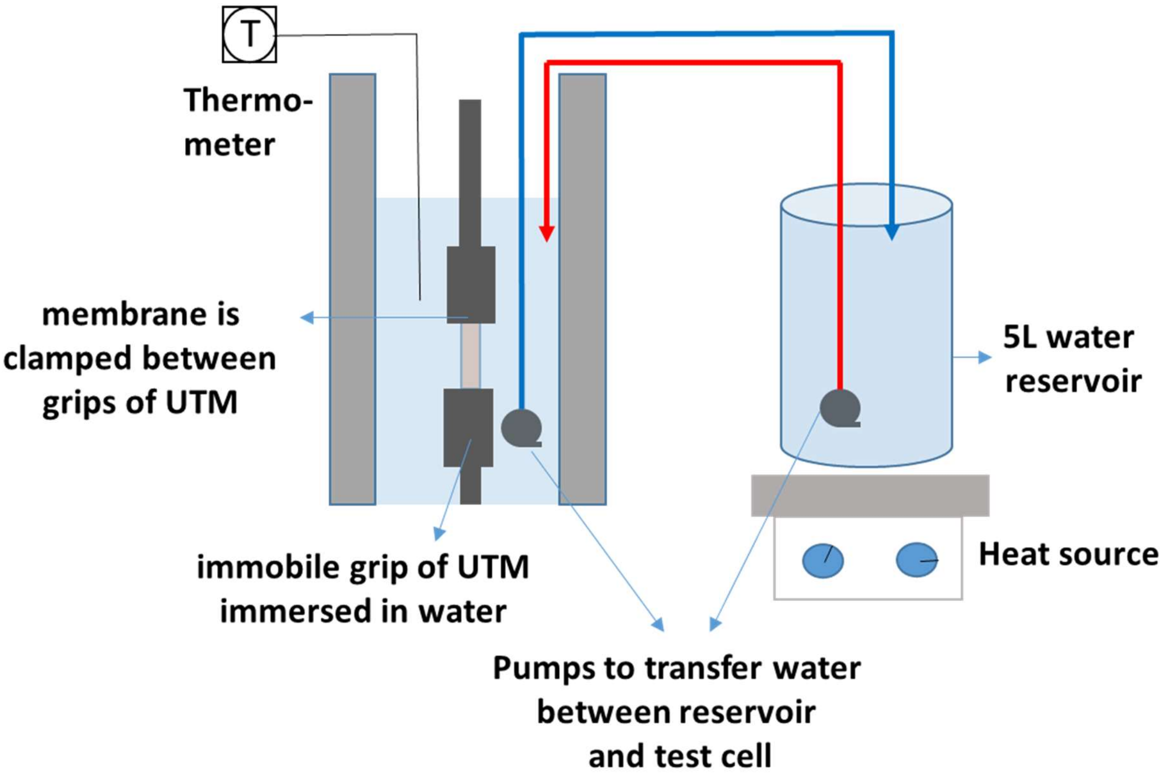

Figure S1: Experimental setup for measuring tensile strength in water at controlled temperature with a Universal Testing Machine (UTM). When the upper grip is moved upwards, buoyancy decreases. Because the grip has a long shaft, the measured load roughly increases linearly by about 0.05 N/cm, and tensile strength values are slightly overestimated. However, the resulting error is small, e.g. 0.5% for a membrane reaching a tensile strength of 10 N and an elongation of 1 cm (ca. 50% elongation).

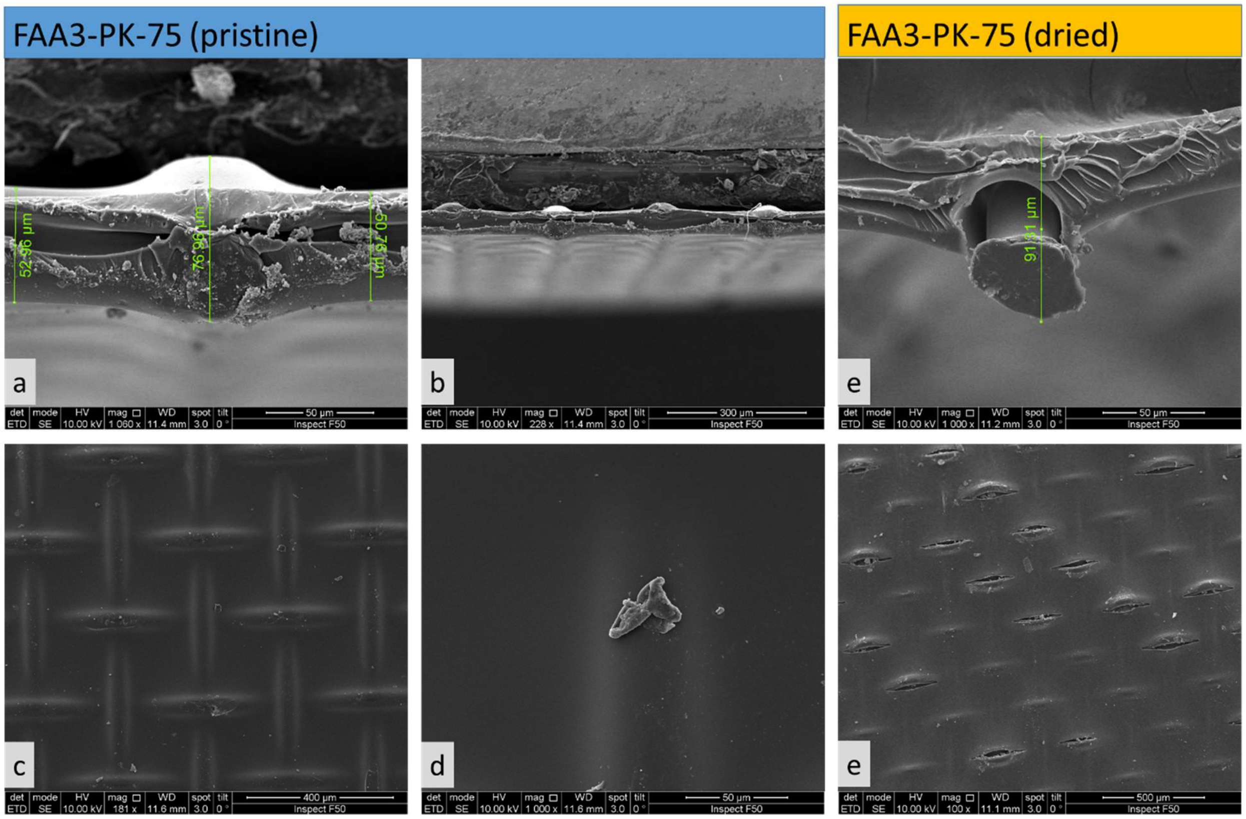

Figure S2: SEM images of FAA3-PK-75. (a)-(d): pristine samples, as received; (e),(f): SEM images taken after drying a sample in the vacuum oven at 60 °C.

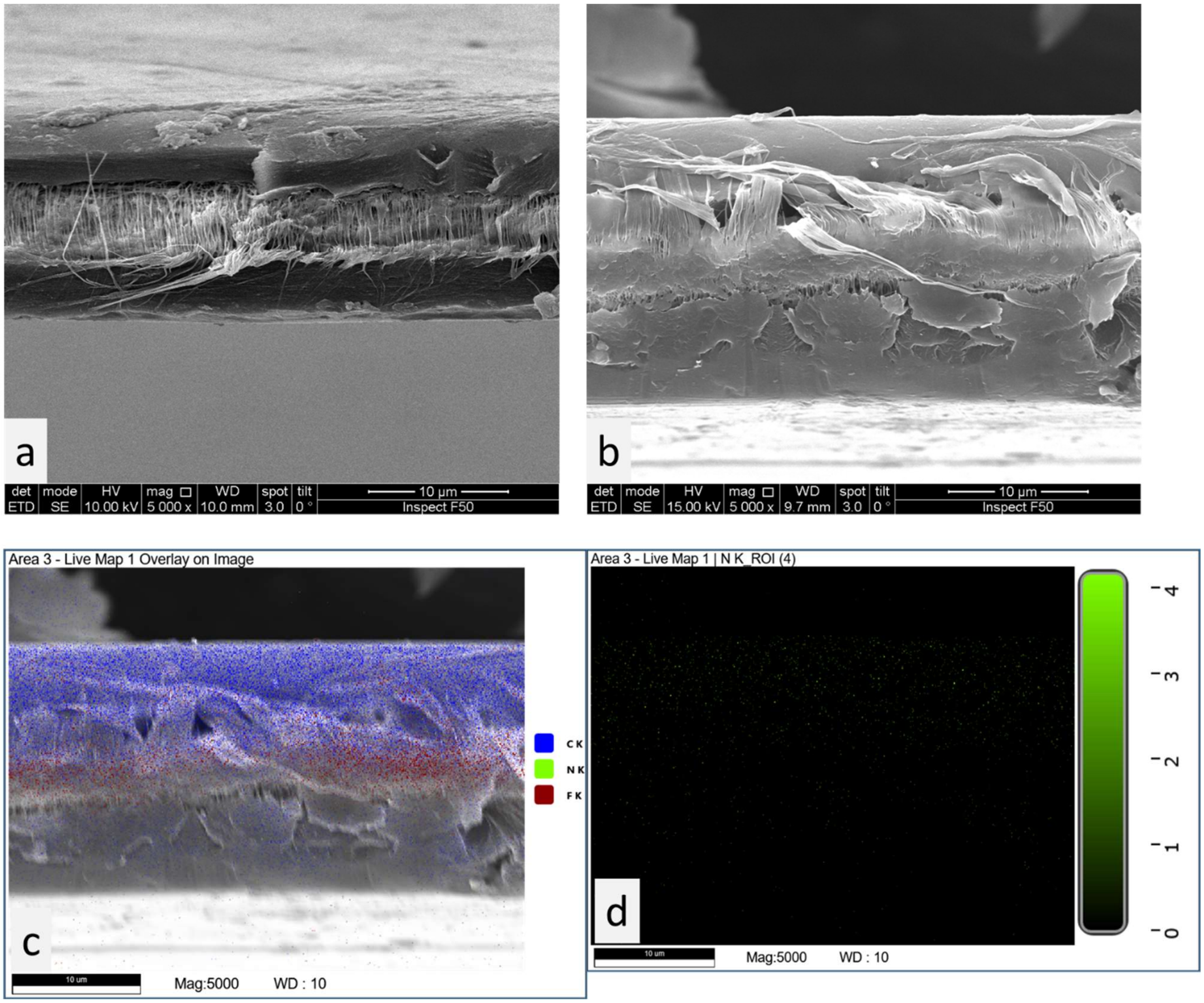

Figure S3: (a) SEM image of a pristine PI-15 membrane, ion exchanged into the chloride form. (b) SEM image of a PI-15 membrane after alkaline degradation shown in Figure 5. (c, d) SEM-EDS pictures of the degraded sample shown in (b).

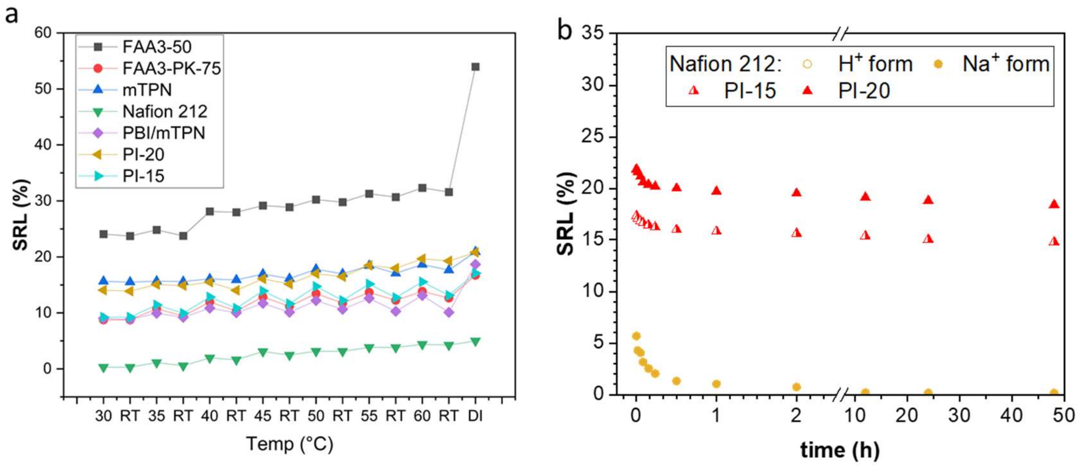

Figure S4: (a) Length swelling for membranes immersed in 1M KOH solution. The temperature was stepwise increased and decreased again to room temperature ("RT"), each step lasted one hour; at the end of the test, the samples were moved from 1M KOH solution into CO_2-free water (CO_2 was removed by bubbling with nitrogen). (b) Length change for hydroxide exchanged membranes which have been stored for 3 hours at 60 °C in 1M KOH and then are put into water which has been equilibrated at room temperature. Dry state dimensions were defined based on membrane samples equilibrated in 1M KOH and then dried in the vacuum at 60 °C.

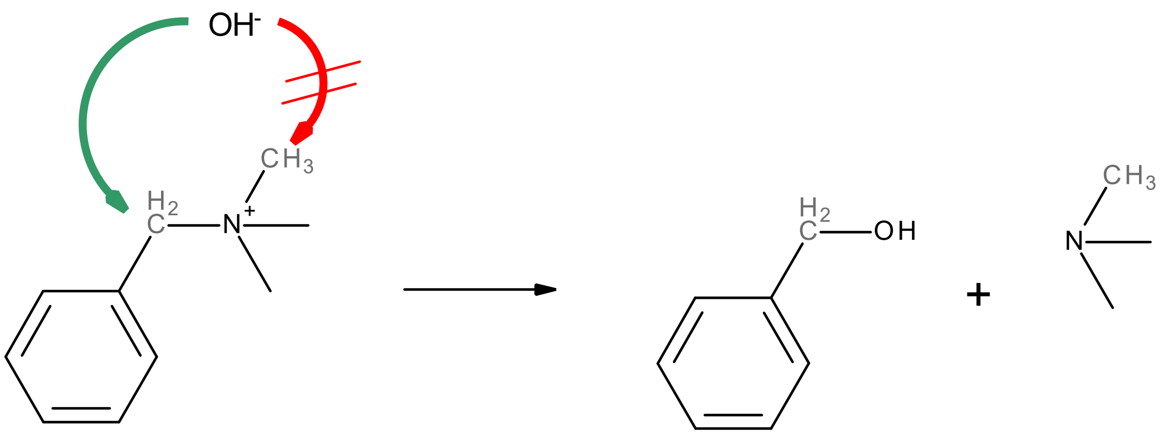

Figure S5: Degradation pathway of the quaternary ammonium groups in FAA3 membranes, based on XPS data shown in Figure 7.

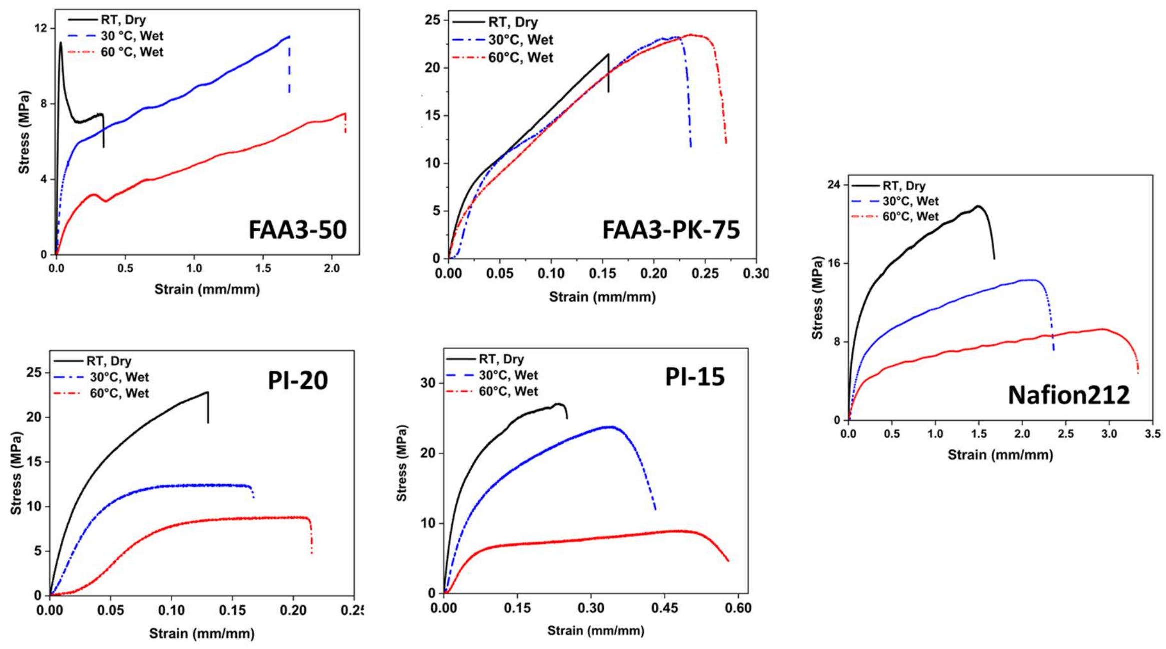

Figure S6: Exemplary stress-strain curves at dry, wet 30 °C and wet 60 °C conditions.
